# Dynamic Pain-Related Changes in Pulse-Graph Measurements in Patients with Primary Dysmenorrhea before and after Electroacupuncture Intervention and Its Correlation with TCM Pattern

**DOI:** 10.1155/2022/3518179

**Published:** 2022-01-27

**Authors:** Yingying Yang, Tianfang Wang, Jian Dong, Ling Tang, Yanping Wang, Ning Li, Lihong Zhao

**Affiliations:** ^1^Department of Diagnostics in Chinese Medicine, School of Chinese Medicine, Beijing University of Chinese Medicine, Beijing 102488, China; ^2^School of Humanities, Beijing University of Chinese Medicine, Beijing 102488, China; ^3^Department of Gynecology, Dongzhimen Hospital, Beijing University of Chinese Medicine, Beijing 100700, China; ^4^School of Acupuncture and Moxibustion, Beijing University of Chinese Medicine, Beijing 102488, China; ^5^Beijing University of Chinese Medicine, Beijing 102488, China

## Abstract

**Objective:**

To explore the dynamic changes recorded in pulse graph related to the changes in the severity of pain before and after electroacupuncture (EA) intervention among young women suffering from primary dysmenorrhea (PD).

**Methods:**

A total of 147 female college students were recruited in this study. Based on participants' symptoms associated with menstruation, they were divided into the PD group and the healthy control group. In addition, participants in the PD group were further sorted into the Cold Coagulation and Blood Stasis Pattern (CCBSP) and Qi Stagnation and Blood Stasis Pattern (QSBSP) based on TCM diagnoses and their pulses differences. Participants in the PD group received EA at maximal pain during menstruation. The primary acupuncture points selected were SP 6 and RN 3, additional RN 4 for CCBSP, and LR 3 for QSBSP. Four observation time points were 7–10 days before menstruation (*T*_0_), maximal pain during menstruation (*T*_1_), immediately after EA (*T*_2_), and 30 mins after EA (*T*_3_). The severity of pain was assessed by a visual analog scale (VAS) along with a pulse analyzer to record the variations of the pulse graph throughout the changes of pain level.

**Results:**

(1) The average VAS score in the PD group decreased from 5.44 ± 1.46 at *T*_1_ to 1.72 ± 1.27 at *T*_2_ and 1.59 ± 1.30 at *T*_3_. The average VAS score in participants of CCBSP at *T*_1_, *T*_2_, and *T*_3_ was higher than that of QSBSP. (2) At *T*_1_, *h*_2_, *h*_3_, *h*_4_, and *w*_1_/*t* were all significantly increased, compared with those at *T*_0_. At *T*_2_, *t* and *t*_5_ were both significantly increased, and *w*_1_/*t*, *t*_1_, and *t*_1_/*t* were all significantly decreased, compared with those at *T*_1_. At *T*_3_, *w*_1_/*t*, *t*_1_, and *t*_1_/*t* were all significantly increased, and *t* and *t*_5_ were both significantly decreased, compared with those at *T*_2_. (3) Comparing the pulse graphs between the healthy control and the PD groups, *h*_1_ was significantly lower at *T*_0_; *w*_1_/*t* was significantly higher at *T*_1_; *t* was significantly higher at *T*_2_; and *t*_1_ and *t*_1_/*t* were both significantly higher at *T*_3_ in PD group. (4) When comparing the pulse graphs between QSBSP and CCBSP, *t*_4_/*t*_5_ was significantly higher at *T*_0_ and *t*_1_ was significantly higher at *T*_1_ in the CCBSP group.

**Conclusion:**

EA is effective in relieving primary dysmenorrhea. Our results showed the opposite changing of the pulse graph recorded before the onset of pain to the maximum pain and that from maximum pain to pain relief. Indeed, there were differences in the recorded pulse graphs between CCBSP and QSBSP (two patterns of PD) as described in traditional Chinese pulses diagnosis. The study has been registered in the Chinese Clinical Trial Registry (registered number: ChiCTR2000040065; registered date: 2020/11/19).

## 1. Introduction

Pulse diagnosis is a unique diagnostic method used in Traditional Chinese Medicine (TCM). In pulse diagnosis, pulse manifestation/picture is obtained, which refers to the perception of pulses felt by the index finger, middle finger, and ring finger along the radial artery of the patient. According to TCM theory, the variations of pulses, appreciated by the practitioner, reflect the conditions of qi and blood [1] and the overall functions of *zang-fu* organs in our body. Pain is a common clinical symptom that can have various underlying causes, that is, dysfunction in *zang-fu* organs, qi, or blood that can be diagnosed through pulse palpation. The ancient TCM textbooks describe that pain-related pulses can be wiry, tight, throbbing, or regularly intermittent. Do these abovementioned pulses correlate to specific disease/symptoms? Whether there are differences in pulses during pain and after pain relief? Whether there is a correlation between the classical description of “patterns, that is, the underlying causes or pathogens” through traditional pulses diagnosis and the changes in characteristics of pulses as recorded by pulse analyzer? In addition, there is a paucity of data regarding the changes in characteristics of pulses associated with the changes in pain level.

Primary dysmenorrhea (PD) is defined as lower abdominal cramp associated with menstruation without pelvic pathology. Acupuncture has been used successfully as a treatment for PD. Recent studies have shown that electroacupuncture (EA) can relieve pain (dredge meridian as described in TCM) without causing apparent adverse effects [2–6]. According to TCM theory, different underlying causes can manifest as the same symptoms. Women suffering from PD can be classified into Cold Coagulation and Blood Stasis Pattern (CCBSP) and Qi Stagnation and Blood Stasis Pattern (QSBSP) based on TCM diagnoses and their pulses differences. Both patterns' manifestation as blood stasis, except the root cause, can be caused by either cold coagulation or qi stagnation.

Given the subjectivity of the pulse manifestation acquired by individual practitioners based on their experiences, there are great interests in the development of pulse analyzer to detect pulse manifestation objectively, and it is increasingly used for the study of pulse manifestation. At present, the most commonly used equipment consists of applying pressure sensors at the radial artery to detect pulse information that simulates traditional pulse diagnosis but objectively. It has been used successfully to replace the traditional pulse diagnosis acquired by individual practitioners. The pulse information is detected by the pressure sensor placed on the radial artery, and the trajectory of the pulse is extracted, that is, the pulse wave graph (referred to as pulse graph). The pulse graph is formed by collecting all the measurements sensed by the pulse analyzer during the heart's contraction and the propagation of the pulse wave along the vasculature to the radial artery. All the curves and inflection points in the pulse graph are then extracted and analyzed based on modern scientific and technological methods. After all the information is extracted, the pulse-graph measurements (including amplitude value and time value) are collected. From the perspective of vascular hemodynamics, the mechanism of pulse changes because of pain can be studied objectively.

We selected PD participants, consisting of underlying CCBSP and QSBSP based on TCM diagnoses and their pulses differences, as experiment model and healthy females without PD as a control to compare and analyze their pulse graph before menstruation, at maximal menstrual pain, immediately after EA, and 30 mins after EA. We also explored the possible correlation between the characteristics of the pulse graph and the traditional pulse diagnosed pattern. The aim of this study is to provide a scientific explanation for traditional TCM pulse diagnosis of disease and underlying causes (i.e., patterns) through objective assessment of radial pulses.

## 2. Methods

### 2.1. Subject

#### 2.1.1. Subject Source

From May 2019 to December 2020, female college students aged between 18 and 30 with PD (CCBSP or QSBSP) and those who were healthy without PD were recruited from Beijing University of Chinese Medicine via WeChat and poster advertisements. Participants were not compensated for participation.

#### 2.1.2. Selection Criteria for PD Participants

① Diagnostic criteria were as follows: the diagnostic was made according to *Primary Dysmenorrhea Clinical Guidelines* released by the Society of Obstetricians and Gynecologists of Canada in 2017. Accordingly, they were patients who had abdominal cramp before or during menstruation with normal ultrasound and pelvic examination [7].
② Pattern differentiation criteria were as follows: the pattern differentiation was made according to the *Guidelines for the Diagnosis and Treatment of Common Gynecological Diseases in Traditional Chinese Medicine* released by the China Society of Traditional Chinese Medicine and Pharmacy in 2012 [8]. According to the TCM diagnosis, the CCBSP presentation consists of abdominal cramp alleviated by warm compress before or during menstruation, dark menstrual blood with clots, usually large amount of thin leucorrhea, a dark tongue with stasis covered with white and greasy coating, and a deep and tight pulse. The QSBSP presentation consists of bloating abdominal cramp worsened by pressure, unsmooth dark menstrual flow with clots, swelling sensation in the breasts, a dark red tongue with stasis covered with thin and white coating, and a wiry pulse. All the PD participants underwent TCM expert's evaluation and were classified into CCBSP and QSBSP to facilitate the operation in our experiment.
③ Inclusion criteria were as follows: females who were aged between 18 and 30 years and had not given birth, who met the abovementioned criteria and their prior three menstruations' pain being greater than or equal to 4 in a 10 VAS; signed informed consent; no skin damage in the left wrist at location radial artery.
④ Exclusion criteria were as follows: patients with the premenstrual syndrome (PMS), who had a common cold or external injury history in recent week or any diseases of the heart, lung, liver, kidney, gastrointestinal system, immune system, or mental disorder; those who were not able to commit to the duration of the study, for example, frequent changes in the working environment or unstable living environment; those who took analgesics or other analgesic therapies 24 hours before the experiment.

#### 2.1.3. Selection Criteria for Healthy Control Population

Healthy females who were between 18 and 30 years and had not given birth were included. In addition, they were able to commit to the entire duration of the study, normal physical examination, and intact skin at the left radial artery distribution at the wrist. Basically, these participants' conditions matched those of PD participants except without any PD symptoms.

#### 2.1.4. Sample Size

The sample size was calculated based on our previous study on *h*_1_ of the recorded pulse graph [9]. With the CCBSP and QSBSP being 16.265 (mm) ±3.511 (*s*_e_) and 13.964 (mm) ±3.227 (*s*_c_), respectively, the maximum sample size measured by this formula was 46 cases (*α* = 0.05; *β* = 0.1). Furthermore, assuming that 15% of cases would be lost to follow-up, 54 cases would be needed for each “pattern” and the healthy control group.

### 2.2. Electroacupuncture Analgesia

Eligible PD participants received EA at maximal pain during menstruation (*T*_1_). Previous bibliometrics study showed that SP 6 (Sanyinjiao) was the most commonly used point for PD, RN 4 (Guanyuan) was added for CCBSP, and LR 3 (Taichong) was added for QSBSP [10]. After discussing with experts in acupuncture, SP 6 (Sanyinjiao) and RN 3 (Zhongji) were selected as the main acupuncture points, with RN 4 (Guanyuan) added for CCBSP, and LR 3 (Taichong) added for QSBSP. SP 6 (Sanyinjiao) is a point of the spleen meridian and the confluent point of the liver, spleen, and kidney meridians; therefore, acupuncture at SP 6 (Sanyinjiao) can relieve dysmenorrhea through regulating qi and promoting blood circulation along with the liver, spleen, and kidney meridians. RN 3 (Zhongji) is a point of the conception vessel. Acupuncture at RN 3 (Zhongji) can relieve dysmenorrhea by warming and nourishing both the thoroughfare vessel and conception vessel through promoting qi and blood circulation. LR3 (Taichong) is a point of the liver meridian. Acupuncture at LR3 (Taichong) can relieve dysmenorrhea by soothing the liver stagnation and promoting qi and blood circulation. RN 4 (Guanyuan) is a point of the conception vessel, and acupuncture at RN 4 (Guanyuan) can relieve dysmenorrhea by warming yang, promoting blood circulation, and strengthening the body [11]. All PD participants were asked to urinate to empty the bladder at first and then lie down in a supine position. After routine disinfection with 75% alcohol on the local skin, Hwato (brand) disposable acupuncture needles (0.25 mm × 40 mm) were inserted perpendicularly at SP 6 (Sanyinjiao), RN 3 (Zhongji), and RN 4 (Guanyuan) about 1 inch, and disposable acupuncture needles (Hwato; 0.25 mm × 25 mm) were inserted perpendicularly at LR3 (Taichong) about 0.5 inches. Following needle insertion, slight, equal manipulations of lifting, twirling, and thrusting were performed until “deqi”; that is, the participants had sensations of distention, soreness, numbness, and heaviness, which is an indication of effective acupuncture treatment. A pair of electrodes from the EA apparatus were then connected to the needle handles at SP 6 and RN 3, respectively. Sparse and dense waves with alternated frequencies of 2 Hz/100 Hz adjust the stimulation intensity of 1 mA to strengthen the stimulation as indicated by mild twitching of the skin around the acupoints without pain. The total duration of EA stimulation was 30 minutes.

### 2.3. Observation Time

All PD participants and healthy control participants were observed at four time points. The data collected at the four time points were matched between the two groups. The four time points are described as follows:

1. PD participants: 7–10 days before menstruation (*T*_0_), maximal pain during menstruation (*T*_1_), immediately after EA (*T*_2_), and 30 mins after EA (*T*_3_). *T*_1_ was defined as the subject reporting that her pain level reached the highest pain level as she experienced in the previous menstrual period.
2. Healthy control population: 7–10 days before menstruation (*T*_0_), the first or second day during menstruation (*T*_1_), 30 mins after *T*_1_ (*T*_2_), and 30 mins after *T*_2_ (*T*_3_).

### 2.4. Assessments

#### 2.4.1. Pain Measurement

Pain intensity of PD participants was assessed at *T*_0_, *T*_1_, *T*_2_, and *T*_3_ using a 10 cm visual analog scale (VAS), a horizontal row with equidistant numbers from 0 representing “no pain” to 10 representing “pain as bad as you can imagine.” The PD participants marked a specific point on VAS based on their pain intensity [12–15].

#### 2.4.2. Pulse-Graph Measurement

**Figure 1:**
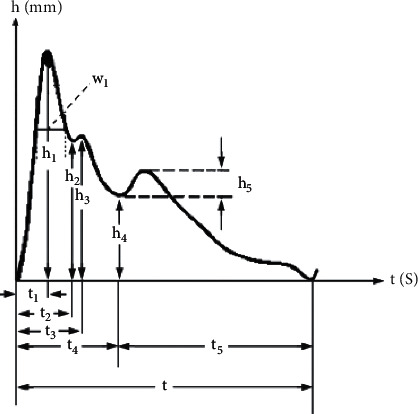
Pulse graph and measurements. *Note. h*_1_: amplitude of the peak of the main wave; *h*_2_: amplitude of the gorge of the main wave; *h*_3_: amplitude of the peak of the prodicrotic wave; *h*_4_: amplitude of the dicrotic notch; *h*_5_: amplitude of the dicrotic wave amplitude; *t*_1_: time of the peak of the main wave; *t*_2_: time of the gorge of the main wave; *t*_3_: time of the peak of the prodicrotic wave; *t*_4_: time of the dicrotic notch; *t*_5_: time of the peak of the dicrotic wave; *t*: time from the starting point to the ending point of the pulse graph; *w*_1_: width of the upper 1/3 of the main wave.

Pulse graph was formed using DS01-C pulse analyzer (Shanghai Machinery Standard 20,202,200,061, Shanghai Daosheng Medical Technology Co., Ltd.). The baroreceptors of the pulse analyzer acquire pulse signals to pulse graph using the computer program. With the time-domain technique, the inflection points in the pulse graph were quantitatively extracted. Then the data recorded in the pulse graph were analyzed, including amplitude value (*h*_1_–*h*_5_), time value (*t*_1_–*t*_5_, *w*_1_, heart rate (HR)), and the relative ratio (*t*_1_/*t*, *w*_1_/*t*, *t*_4_/*t*_5_), to gain a better understanding of the underlying cardiovascular condition [16]. The description of the pulse graph is shown in Figure 1.

The pulse measurements are described as follows: *h*_1_ mainly reflects the left ventricular ejection function and arterial elasticity. *h*_2_, *h*_3_, and *h*_4_ mainly reflect the peripheral resistance. *h*_5_ mainly reflects the arterial elasticity and the function of the aortic valve. *w*_1_ and *w*_1_/*t* mainly reflect the duration sustained by the highest level of intra-arterial pressure. *t*_1_–*t*_5_ are the time values of the corresponding amplitudes; specifically, *t*_1_ corresponds to the rapid ejection phase of the left ventricular; *t*_4_ corresponds to the left ventricular systole; *t*_5_ corresponds to the left ventricular diastole; *t*_2_ and *t*_3_ are usually not discussed; *t* corresponds to a cardiac cycle. *t*_1_/*t* mainly reflects the contractility of the heart. *Cunkou* (the region where the radial artery can be felt near the styloid process of radius) is the position for pulse diagnosis in TCM. In TCM, *cunkou* is divided into three sections, namely, *cun*, *guan*, and *chi*, which, respectively, correspond to the heart, liver, and kidney, three main organs that can reflect the physical condition of a person. We chose the *guan* section in the left *cunkou* for applying the pulse analyzer.

After 10 mins of rest, the subjects were asked to sit comfortably, breath naturally, and bend the left elbow with palm upwards to keep the radial artery of the upper limb at the same level as the heart. A sensor was fixed at the *guan* section, while the subjects were sitting still. The pulse analyzer collected pulse graph with different pressures, and then the computer program automatically selected the best pulse graph for analysis; all data collected were transmitted and stored in a computer.

**Figure 2:**
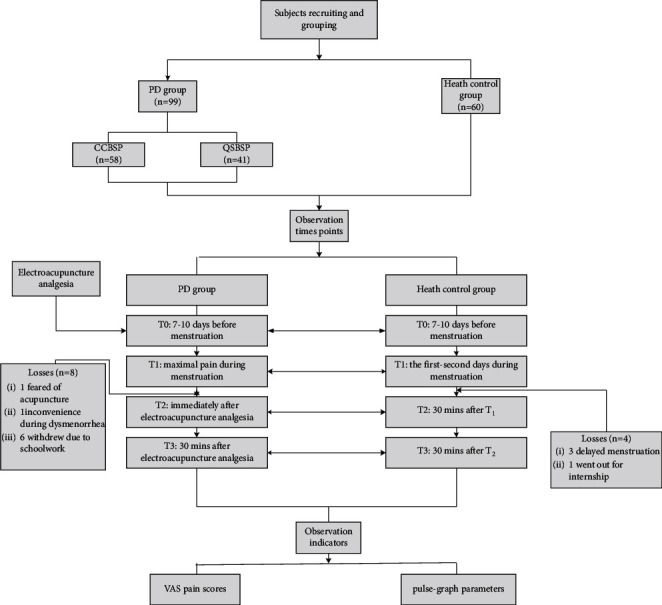
Flowchart.

Figure 2 shows the study flowchart.

### 2.5. Statistical Analysis

Repeated measure analysis of variance was conducted with SPSS 20.0 to assess the data at four observation points. If the Huynh-Feldt condition was met (spherical test *P* > 0.05), the unary variance analysis result was used. Otherwise, multivariate analysis of variance or mixed model results would be used. Measurement data were presented as mean ± standard. Moreover, the difference with *P* < 0.05 was considered statistically significant.

## 3. Results

### 3.1. Study Population

We approached 211 college students in total for the study, 30 college students refused to participate in the study, then 22 college students were excluded because they did not meet the inclusion criteria, and additional 12 college students were lost to follow-up.

Since QSBSP was relatively rare in clinical practice, only 37 cases were included in our study. Therefore, only 91 PD participants (CCBSP (*n* = 54) and QSBSP (*n* = 37)) and 56 health controls were included in the final analyses. All 147 participants received pelvic examination and ultrasound to rule out pelvic pathology prior to enrolling in the study. The mean age of PD participants was 20.55 ± 2.68 years old, of which the CCBSP was 20.65 ± 2.99 years old, and the QSBSP was 20.41 ± 2.19 years old. The mean age of the healthy control population was 21.23 ± 3.05 years old.

### 3.2. Pain Situation

#### 3.2.1. Severity of Pain Experienced by PD Participants

**Figure 3:**
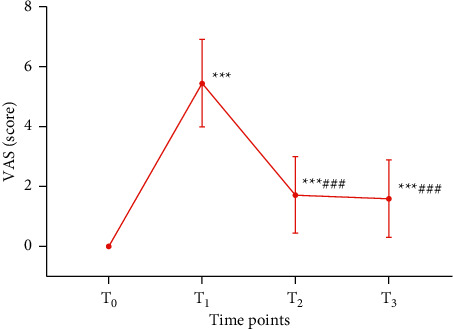
VAS score changes at four time points in 91 participants with PD. *Note. T*_0_ means 7–10 days before menstruation, *T*_1_ refers to the time with maximal pain during menstruation, *T*_2_ refers to the time immediately after EA, and *T*_3_ means 30 mins after EA. ^*∗∗∗*^*P* < 0.001, compared with *T*_0_; ^###^*P* < 0.001, compared with *T*_1_.

At *T*_0_, none of the PD participants had pain (Figure 3).

At *T*_1_ (maximum pain), the VAS score was 5.44 ± 1.46 (range, 3.5 to 10). At *T*_2_ (immediately after EA), the VAS score was 1.72 ± 1.27 (range, 0 to 4.6). At *T*_3_ (30 mins after EA), the VAS score was 1.59 ± 1.30 (range, 0 to 4.1). The VAS scores at *T*_1_, *T*_2_, and *T*_3_ were all significantly increased compared with those at *T*_0_ (*P* < 0.001), and the scores at *T*_2_ and *T*_3_ were significantly decreased compared with those at *T*_1_ (*P* < 0.001), but the pain scores between *T*_2_ and *T*_3_ were not significantly different (*P* > 0.05).

#### 3.2.2. Comparison of Pain Severity between CCBSP and QSBSP

**Figure 4:**
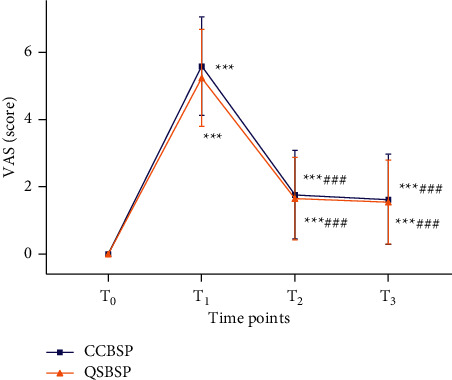
Comparison of VAS scores between CCBSP and QSBSP. *Note.*^*∗∗∗*^*P* < 0.001, compared with *T*_0_; ^###^*P* < 0.001, compared with *T*_1_.

Figure 4 shows the two patterns of CCBSP and QSBSP of PD participants' pain scores. As shown before, at *T*_0_, no pain was experienced in either group of participants. At *T*_1_, CCBSP and QSBSP participants reported that the VAS scores were 5.58 ± 1.46 (range, 4 to 10) and 5.24 ± 1.44 (range, 3.5 to 10), respectively. At *T*_2_, the VAS scores were 1.76 ± 1.31 (range, 0 to 4.1) and 1.65 ± 1.221 (range, 0 to 4.6), respectively. At *T*_3_, the VAS scores were 1.62 ± 1.34 (range, 0 to 3.9) and 1.54 ± 1.251 (range, 0 to 4.1), respectively. Although CCBSP participants had slightly higher pain scores than those of the QSBSP group at *T*_1_, *T*_2_, and *T*_3_, none of them reached statistical significance (*P* > 0.05).

### 3.3. Changes in Pulse-Graph Measurements

#### 3.3.1. Changes in Pulse Graph of PD Participants during the Study Period

**Figure 5:**
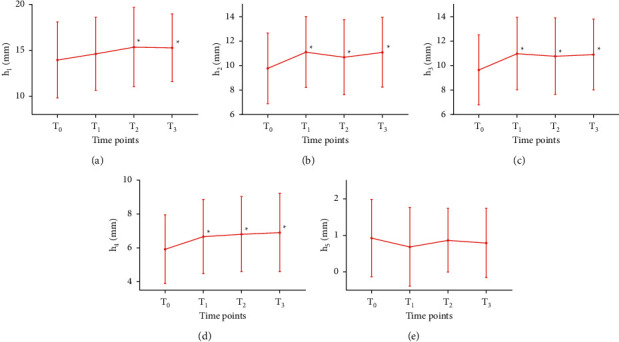
Amplitudes changes in pulse graph (*h*) at four time points in 91 PD participants. *Note.*^*∗*^*P* < 0.05, compared with *T*_0_.

As shown in Figure 5 (for the statistical data, see Table 1 in the supplementary material), *h*_2_, *h*_3_, and *h*_4_ were all significantly increased at *T*_1_ (*P* < 0.05) as compared with *T*_0_, except *h*_5_ was decreased at *T*_1_ as compared with *T*_0_ but did not reach statistical significance (*P* > 0.05). At *T*_2_, *T*_3_, *h*_1_, *h*_2_, *h*_3_, and *h*_4_ were all significantly increased, compared with those at *T*_0_ (*P* < 0.05).

**Figure 6:**
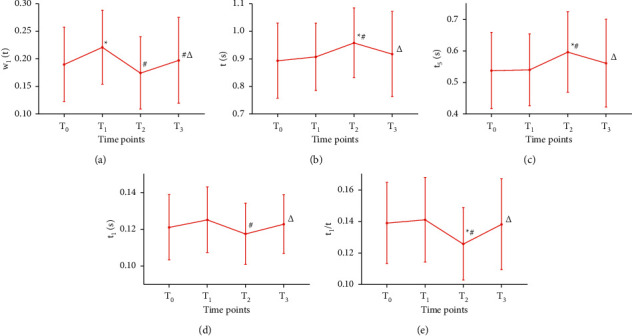
Times changes in pulse-graph measurements (*t*) at four time points in 91 PD participants. *Note.*^*∗*^*P* < 0.05, compared with *T*_0_; ^#^*P* < 0.05, compared with *T*_1_; ^△^*P* < 0.05, compared with *T*_2_.

As shown in Figure 6 (for the statistical data, see Table 2 in the supplementary material), at *T*_1_, *w*_1_/*t* was significantly increased compared with that at *T*_0_ (*P* < 0.05). At *T*_2_, *t* and *t*_5_ were significantly increased (*P* < 0.05), and *w*_1_/*t*, *t*_1_, and *t*_1_/*t* were significantly decreased compared with those at *T*_1_ (*P* < 0.05). At *T*_3_, *w*_1_/*t*, *t*_1_, and *t*_1_/*t* were significantly increased (*P* < 0.05), and *t* and *t*_5_ were significantly decreased compared with those at *T*_2_ (*P* < 0.05). At *T*_2_, *t* and *t*_5_ were significantly increased (*P* < 0.05), and *t*_1_/*t* was significantly decreased compared with those at *T*_0_ (*P* < 0.05). There were no significant differences between other time points.

**Figure 7:**
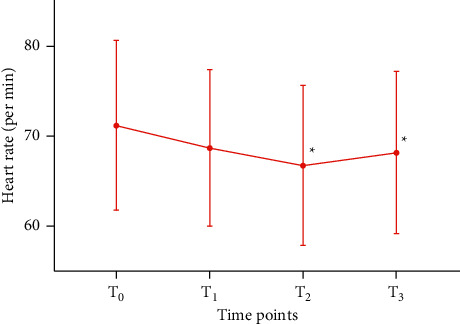
Changes in HR at four time points in 91 PD participants. *Note.*^*∗*^*P* < 0.05, compared with *T*_0_.

Changes in heart rate (HR) at four time points in 91 participants with PD were shown in Figure 7 (for the statistical data, see Table 3 in the supplementary material). HR at *T*_1_ was decreased compared with that at *T*_0_ (*P* > 0.05), decreased at *T*_2_ compared with that at *T*_1_ (*P* > 0.05), and increased at *T*_3_ compared with that at *T*_2_ (*P* > 0.05). At both *T*_2_ and *T*_3_, HR was significantly decreased compared with that at *T*_0_ (*P* < 0.05).

#### 3.3.2. Comparison of Changes in Pulse-Graph Measurements between the PD Group and the Healthy Control Group

**Figure 8:**
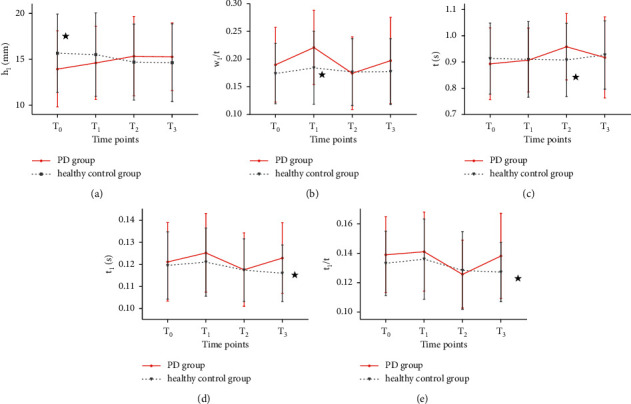
Comparison of changes in pulse-graph measurements between PD group and healthy control group at four time points. *Note.*^★^*P* < 0.05, compared with PD group.

As shown in Figure 8 (for the statistical data, see Table 4 in the supplementary material), at *T*_0_, *h*_1_ of the PD group was significantly lower than that of the healthy control group (*P* < 0.05); at *T*_1_, *w*_1_/*t* was significantly higher than that of the healthy control group (*P* < 0.05). At *T*_2_, *t* was significantly higher than that of the healthy control group (*P* < 0.05). At *T*_3_, *t*_1_ and *t*_1_/*t* were significantly higher than those of the healthy control group (*P* < 0.05). There were no significant differences at other time points between the two groups.

#### 3.3.3. Comparison of Changes in Pulse-Graph Measurements between CCBSP and QSBSP

**Figure 9:**
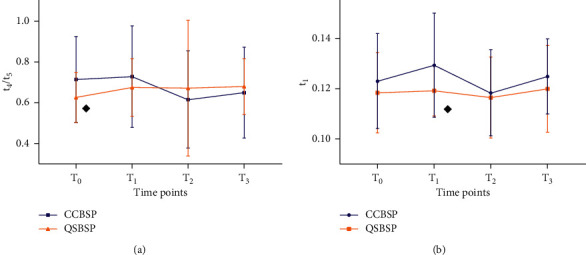
Comparison of changes in pulse-graph measurements between CCBSP and QSBSP at four time points. *Note.*^◆^*P* < 0.05, compared with CCBSP.

As shown in Figure 9 (for the statistical data, see Table 5 in the supplementary material), at *T*_0_, *t*_4_/*t*_5_ of CCBSP was significantly higher than that of QSBSP (*P* < 0.05). At *T*_1_, *t*_1_ of CCBSP was significantly higher than that of QSBSP (*P* < 0.05). There were no significant differences at other time points between the two patterns.

## 4. Discussion

The etiology of PD is not precisely understood, but most women suffering from PD have increased production of endometrial prostaglandin during menstruation without any identifiable pelvic pathology. Prostaglandins may cause ischemia by acting on the myometrium and blood vessels of the uterus to promote contraction of uterine arteries and smooth muscles, thus manifesting the following symptoms: abdominal cramp, nausea, and vomiting. In TCM, it is believed that PD is caused by blood stasis [17, 18]. Similar to previous studies, we also demonstrated that EA could reduce the pain experienced by all PD participants, regardless of the TCM diagnoses in this study. In addition, we found the changes in the pulse graph as detected by the pulse analyzer.

The pulse-graph measurements of PD participants showed dynamic changes in the whole study period, both before and after EA intervention. At *T*_0_, *h*_1_ was significantly lower than that of the healthy control population. It indicates that the left ventricular ejection function of PD participants is significantly lower than that of the healthy control population before the onset of dysmenorrhea. As a result, the pulse tendency of PD participants in the pulse-graph measurements is low, flat, and weak, and it corresponds to the TCM diagnosis of a deficiency pulse [19], which can be interpreted by TCM theory that PD participants may have qi-blood deficiency. At *T*_1_, *w*_1_/*t* of PD participants was significantly higher than that of the healthy control population; besides, *h*_2_, *h*_3_, and *h*_4_ and *w*_1_/*t* were all significantly increased from *T*_0_ to *T*_1_. Therefore, we can conclude that pain could be caused by a decrease in the arterial elasticity, an increase in the peripheral resistance, and a longer duration of the highest level of intra-arterial pressure. This phenomenon is consistent with the wiry pulse in TCM associated with the pain [20], and the results of this study are consistent with the results of previous studies [21]. At *T*_2_, *t* of PD participants was significantly higher than that of the healthy control population, *t* and *t*_5_ were both significantly increased, and *w*_1_/*t*, *t*_1_, and *t*_1_/*t* were significantly decreased from *T*_1_ to *T*_2_. Accordingly, it is concluded that pain relief could increase the left ventricular ejection function and the left ventricular diastole and cardiac cycle and decrease the duration maintained by the highest level of intra-arterial pressure and the rapid ejection phase of the left ventricular. This phenomenon is consistent with the loose pulse in TCM. There are almost opposite pulses in terms of vascular tension: the wiry pulse and loose pulse. The wiry pulse is tense, while the loose pulse is sluggish, soft, and relatively slow. So the loose pulse is more common when the pain is relieved, at which time the smooth flow of qi and blood is restored [22, 23].

The results of this study also demonstrated that there were differences in the pulse graph between CCBSP and QSBSP. At *T*_0_, *t*_4_/*t*_5_ of CCBSP participants was significantly higher than that of QSBSP. It indicates that the left ventricular systole in CCBSP is significantly higher than that of QSBSP before dysmenorrhea. The possible reason for this phenomenon based on TCM might be that the symptoms are the manifestation of the impeded flow of qi and blood of CCBSP, while the pulse graph is manifested as a decrease in contractility of the heart and prolonged left ventricular systole. At *T*_1_, *t*_1_ of CCBSP participants was significantly higher than that of QSBSP. It indicates that the ejection function of the heart of CCBSP is significantly declined compared to that of QSBSP participants. The possible reason for this phenomenon may be that the severity of pain experienced by CCBSP participants is slightly higher than that of the QSBSP participants during menstruation, resulting in blood vessel constriction and a decreased ejection fraction of the heart.

## 5. Conclusion

EA intervention could significantly relieve the pain in PD participants, and the pain was able to be captured by an objective pulse analyzer. In addition, there were differences in the pulse graphs before and after dysmenorrhea between the PD participants and the healthy population. We also demonstrated that there were differences between two patterns of PD based on TCM diagnoses, and their pulses differences were also captured by the pulse analyzer. This is the first documentation that TCM diagnosed two different patterns of PD indeed having differences in pulse manifestations.

## Data Availability

Original data can be obtained from Tables 1to 5 in Supplementary Materials.

## Supplementary Materials

Supplementary Materials A concise description of the supplementary material (Tables 1–5): Table 1 describes the amplitudes changes in pulse graph (*h*) at four time points in 91 PD participants and corresponds to Figure 5. Table 2 describes the time changes in pulse-graph measurements (*t*) at four time points in 91 PD participants and corresponds to Figure 6. Table 3 describes the changes in HR at four time points in 91 PD participants and corresponds to Figure 7. Table 4 describes the comparison of changes in pulse-graph measurements between the PD group and the healthy control group at four time points and corresponds to Figure 8. Table 5 describes the comparison of changes in pulse-graph measurements between CCBSP and QSBSP at four time points and corresponds to Figure 9.
