# Supplementary material for: Dynamic Pain-Related Changes in Pulse-Graph Measurements in Patients with Primary Dysmenorrhea before and after Electroacupuncture Intervention and Its Correlation with TCM Pattern
Source: Evid Based Complement Alternat Med. 2022 Jan 27;2022:3518179. doi: 10.1155/2022/3518179 (PMC8813248; doi:10.1155/2022/3518179)
Supplement: Supplementary Materials — A concise description of the supplementary material (Tables 1–5): Table 1 describes the amplitudes changes in pulse graph (h) at four time points in 91 PD participants and corresponds to Figure 5. Table 2 describes the time changes in pulse-graph measurements (t) at four time points in 91 PD participants and corresponds to Figure 6. Table 3 describes the changes in HR at four time points in 91 PD participants and corresponds to Figure 7. Table 4 describes the comparison of changes in pulse-graph measurements between the PD group and the healthy control group at four time points and corresponds to Figure 8. Table 5 describes the comparison of changes in pulse-graph measurements between CCBSP and QSBSP at four time points and corresponds to Figure 9. [file 3518179.f1.doc]

**supplementary material (Table 1-Table 5)**

A concise description of the supplementary material (Table 1-Table 5):

Table 1 describes the amplitudes changes in pulse-graph (h) at four time points in 91 PD participants, and corresponds to Figure 5.

Table 2 describes the times changes in pulse-graph measurements (t) at four time points in 91 PD participants, and corresponds to Figure 6.

Table 3 describes the changes in HR at four time points in 91 PD participants, and corresponds to Figure 7.

Table 4 describes the comparison of changes in pulse-graph measurements between PD group and healthy control group at four time points, and corresponds to Figure 8.

Table 5 describes the comparison of changes in pulse-graph measurements between CCBSP and QSBSP at four time points , and corresponds to Figure 9.

| Table 1 Amplitudes changes in pulse-graph (h) at four time points in 91 PD participants (`x±s) | | | | | | | | | | | |
| --- | --- | --- | --- | --- | --- | --- | --- | --- | --- | --- | --- |
|  |  | Observation Time | | | | Time effect | | Interaction  effect | | Group effect | |
| Items(units) | Groups(Cases) | T_0_ | T_1_ | T_2_ | T_3_ | *F* | *P* | *F* | *P* | *F* | *P* |
| h_1_(mm) | PD group（n=91） | 13.95±4.15 | 14.62±4.00 | 15.35±4.32^*^ | 15.27±3.69^*^ | 0.192 | 0.901 | 6.13 | 0.001 | 0.35 | 0.555 |
| h_2_(mm) | PD group（n=91） | 9.77±2.90 | 11.11±2.90^*^ | 10.68±3.06^*^ | 11.08±2.85^*^ | 2.879 | 0.038 | 4.479 | 0.005 | 0.255 | 0.614 |
| h_3_(mm) | PD group（n=91） | 9.64±2.86 | 10.98±2.96^*^ | 10.76±3.13^*^ | 10.90±2.90^*^ | 3.345 | 0.019 | 4.986 | 0.002 | 0.711 | 0.401 |
| h_4_(mm) | PD group（n=91） | 5.91±2.03 | 6.66±2.20^*^ | 6.80±2.22^*^ | 6.90±2.32^*^ | 1.302 | 0.276 | 4.619 | 0.004 | 0.691 | 0.407 |
| h_5_(mm) | PD group（n=91） | 0.93±1.06 | 0.68±1.08 | 0.86±0.88 | 0.79±0.95 | 1.928 | 0.128 | 0.717 | 0.543 | 5.441 | 0.021 |
| Notes: PD group: 7-10 days before menstruation (T_0_), maximal pain during menstruation (T_1_), immediately after acupuncture analgesia (T_2_), and 30 mins after acupuncture analgesia(T_3_).  *P<0.05, compared with T_0_. | | | | | | | | | | | |

| Table 2 Times changes in pulse-graph measurements (t) at four time points in all PD participants (`x±s) | | | | | | | | | | | | | | | | |
| --- | --- | --- | --- | --- | --- | --- | --- | --- | --- | --- | --- | --- | --- | --- | --- | --- |
|  |  | Observation Time | | | | | | | Time effect | | | Interaction  effect | | | Group effect | |
| Items(units) | Groups(Cases) | T0 | | T1 | | T2 | | T3 | F | | P | F | | P | F | P |
| t_1_(s) | PD group（n=91） | 0.12±0.02 | 0.13±0.02 | | 0.12±0.02^#^ | | 0.12±0.02^△^ | | 3.805 | 0.012 | | 3.18 | 0.026 | | 2.625 | 0.107 |
| t_2_(s) | PD group（n=91） | 0.21±0.03 | 0.21±0.03 | | 0.21±0.02 | | 0.21±0.03 | | 0.523 | 0.667 | | 0.562 | 0.64 | | 0.528 | 0.469 |
| t_3_(s) | PD group（n=91） | 0.24±0.04 | 0.23±0.03 | | 0.24±0.04 | | 0.24±0.04 | | 0.031 | 0.862 | | 0.137 | 0.938 | | 2.218 | 0.139 |
| t_4_(s) | PD group（n=91） | 0.35±0.04 | 0.36±0.06 | | 0.36±0.07 | | 0.35±0.05 | | 3.933 | 0.01 | | 0.124 | 0.946 | | 3.581 | 0.06 |
| t_5_(s) | PD group（n=91） | 0.54±0.12 | 0.54±0.11 | | 0.60±0.13^*#^ | | 0.56±0.14^△^ | | 3.516 | 0.015 | | 4.422 | 0.004 | | 0.709 | 0.401 |
| t(s) | PD group（n=91） | 0.89±0.14 | 0.91±0.12 | | 0.96±0.13^*#^ | | 0.92±0.16^△^ | | 3.09 | 0.03 | | 4.37 | 0.01 | | 0.055 | 0.815 |
| t_1_/t(-) | PD group（n=91） | 0.14±0.03 | 0.14±0.03 | | 0.13±0.02^*#^ | | 0.14±0.03^△^ | | 8.828 | 0 | | 2.806 | 0.039 | | 2.253 | 0.136 |
| t_4_/t_5_(-) | PD group（n=91） | 0.68±0.19 | 0.71±0.21 | | 0.64±0.28 | | 0.66±0.19 | | 2.762 | 0.044 | | 1.219 | 0.305 | | 4.558 | 0.034 |
| w_1_(s) | PD group（n=91） | 0.17±0.06 | 0.20±0.07 | | 0.16±0.07 | | 0.18±0.07 | | 1.938 | 0 | | 1.479 | 0.184 | | 1.891 | 0.155 |
| w_1_/t(-) | PD group（n=91） | 0.19±0.07 | 0.22±0.07^*^ | | 0.17±0.07^#^ | | 0.20±0.08^#△^ | | 7 | 0 | | 3.645 | 0.014 | | 4.797 | 0.03 |
| Notes: PD group: 7-10 days before menstruation (T_0_), maximal pain during menstruation (T_1_), immediately after acupuncture analgesia (T_2_), and 30 mins after acupuncture analgesia(T_3_).  *P<0.05, compared with T_0_; #P< 0.05, compared with T_1_; △P<0.05, compared with T_2_. | | | | | | | | | | | | | | | | |

|  |  | Observation Time | | | | | | | Time effect | | | Interaction  effect | | | Group effect | |
| --- | --- | --- | --- | --- | --- | --- | --- | --- | --- | --- | --- | --- | --- | --- | --- | --- |
| Items(units) | Groups(Cases) | T_0_ | | T_1_ | | T_2_ | | T_3_ | *F* | | *P* | *F* | | *P* | *F* | *P* |
| HR(次/min) | PD group（n=91） | 71.19±9.44 | 68.68±8.71 | | 66.74±8.90^*^ | | 68.16±9.03^*^ | | 4.871 | 0.004 | | 2.807 | 0.048 | | 0.009 | 0.923 |
| Notes: PD group: 7-10 days before menstruation (T_0_), maximal pain during menstruation (T_1_), immediately after acupuncture analgesia (T_2_), and 30 mins after acupuncture analgesia(T_3_).  Notes: *P<0.05, compared with T_0_. | | | | | | | | | | | | | | | | |

Table 3 Changes in HR at four time points in all PD participants (`x±s)

| Table 4 Comparison of changes in pulse-graph measurements between PD group and healthy control group at four time points (`x±s) | | | | | | | | | | | |
| --- | --- | --- | --- | --- | --- | --- | --- | --- | --- | --- | --- |
|  |  | Observation Time | | | | Time effect | | Interaction  effect | | Group effect | |
| Items(units) | Groups(Cases) | T_0_ | T_1_ | T_2_ | T_3_ | *F* | *P* | *F* | *P* | *F* | *P* |
| h_1_(mm) | PD group（n=91） | 13.95±4.15 | 14.62±4.00 | 15.35±4.32 | 15.27±3.69 | 0.192 | 0.901 | 6.13 | 0.001 | 0.35 | 0.555 |
|  | healthy control group（n=56） | 15.67±4.26★ | 15.52±4.54 | 14.71±4.14 | 14.64±4.23 |  |  |  |  |  |  |
| h_2_(mm) | PD group（n=91） | 9.77±2.90 | 11.11±2.90 | 10.68±3.06 | 11.08±2.85 | 2.879 | 0.038 | 4.479 | 0.005 | 0.255 | 0.614 |
|  | healthy control group（n=56） | 10.76±3.05 | 10.78±3.24 | 10.10±2.74 | 10.19±2.94 |  |  |  |  |  |  |
| h_3_(mm) | PD group（n=91） | 9.64±2.86 | 10.98±2.96 | 10.76±3.13 | 10.90±2.90 | 3.345 | 0.019 | 4.986 | 0.002 | 0.711 | 0.401 |
|  | healthy control group（n=56） | 10.38±3.03 | 10.54±3.23 | 9.98±2.65 | 9.99±2.93 |  |  |  |  |  |  |
| h_4_(mm) | PD group（n=91） | 5.91±2.03 | 6.66±2.20 | 6.80±2.22 | 6.90±2.32 | 1.302 | 0.276 | 4.619 | 0.004 | 0.691 | 0.407 |
|  | healthy control group（n=56） | 6.48±2.20 | 6.42±2.14 | 6.14±2.06 | 6.26±2.18 |  |  |  |  |  |  |
| h_5_(mm) | PD group（n=91） | 0.93±1.06 | 0.68±1.08 | 0.86±0.88 | 0.79±0.95 | 1.928 | 0.128 | 0.717 | 0.543 | 5.441 | 0.021 |
|  | healthy control group（n=56） | 1.21±0.97 | 1.07±0.77 | 1.06±0.84 | 1.14±1.00 |  |  |  |  |  |  |
| t_1_(s) | PD group（n=91） | 0.12±0.02 | 0.13±0.02 | 0.12±0.02 | 0.12±0.02 | 3.805 | 0.012 | 3.18 | 0.026 | 2.625 | 0.107 |
|  | healthy control group（n=56） | 0.12±0.02 | 0.12±0.02 | 0.12±0.02 | 0.12±0.02★ |  |  |  |  |  |  |
| t_2_(s) | PD group（n=91） | 0.21±0.03 | 0.21±0.03 | 0.21±0.02 | 0.21±0.03 | 0.523 | 0.667 | 0.562 | 0.64 | 0.528 | 0.469 |
|  | healthy control group（n=56） | 0.20±0.02 | 0.21±0.02 | 0.21±0.02 | 0.20±0.02 |  |  |  |  |  |  |
| t_3_(s) | PD group（n=91） | 0.24±0.04 | 0.23±0.03 | 0.24±0.04 | 0.24±0.04 | 0.031 | 0.862 | 0.137 | 0.938 | 2.218 | 0.139 |
|  | healthy control group（n=56） | 0.23±0.03 | 0.23±0.03 | 0.23±0.03 | 0.23±0.03 |  |  |  |  |  |  |
| t_4_(s) | PD group（n=91） | 0.35±0.04 | 0.36±0.06 | 0.36±0.07 | 0.35±0.05 | 3.933 | 0.01 | 0.124 | 0.946 | 3.581 | 0.06 |
|  | healthy control group（n=56） | 0.34±0.04 | 0.35±0.04 | 0.35±0.04 | 0.33±0.05 |  |  |  |  |  |  |
| t_5_(s) | PD group（n=91） | 0.54±0.12 | 0.54±0.11 | 0.60±0.13 | 0.56±0.14 | 3.516 | 0.015 | 4.422 | 0.004 | 0.709 | 0.401 |
|  | healthy control group（n=56） | 0.57±0.12 | 0.56±0.12 | 0.56±0.13 | 0.59±0.12 |  |  |  |  |  |  |
| t(s) | PD group（n=91） | 0.89±0.14 | 0.91±0.12 | 0.96±0.13 | 0.92±0.16 | 3.09 | 0.03 | 4.37 | 0.01 | 0.055 | 0.815 |
|  | healthy control group（n=56） | 0.91±0.14 | 0.91±0.14 | 0.91±0.14★ | 0.93±0.13 |  |  |  |  |  |  |
| t_1_/t(-) | PD group（n=91） | 0.14±0.03 | 0.14±0.03 | 0.13±0.02 | 0.14±0.03 | 8.828 | 0 | 2.806 | 0.039 | 2.253 | 0.136 |
|  | healthy control group（n=56） | 0.13±0.02 | 0.14±0.03 | 0.13±0.03 | 0.13±0.02★ |  |  |  |  |  |  |
| t_4_/t_5_(-) | PD group（n=91） | 0.68±0.19 | 0.71±0.21 | 0.64±0.28 | 0.66±0.19 | 2.762 | 0.044 | 1.219 | 0.305 | 4.558 | 0.034 |
|  | healthy control group（n=56） | 0.61±0.14 | 0.64±0.13 | 0.66±0.24 | 0.59±0.14 |  |  |  |  |  |  |
| w_1_(s) | PD group（n=91） | 0.17±0.06 | 0.20±0.07 | 0.16±0.07 | 0.18±0.07 | 1.938 | 0 | 1.479 | 0.184 | 1.891 | 0.155 |
|  | healthy control group（n=56） | 0.16±0.06 | 0.17±0.07 | 0.16±0.05 | 0.16±0.06 |  |  |  |  |  |  |
| w_1_/t(-) | PD group（n=91） | 0.19±0.07 | 0.22±0.07 | 0.17±0.07 | 0.20±0.08 | 7 | 0 | 3.645 | 0.014 | 4.797 | 0.03 |
|  | healthy control group（n=56） | 0.17±0.05 | 0.18±0.07★ | 0.18±0.06 | 0.18±0.06 |  |  |  |  |  |  |
| HR(次/min) | PD group（n=91） | 71.19±9.44 | 68.68±8.71 | 66.74±8.90 | 68.16±9.03 | 4.871 | 0.004 | 2.807 | 0.048 | 0.009 | 0.923 |
| HR(次/min) | PD group（n=91） | 71.19±9.44 | 68.68±8.71 | 66.74±8.90 | 68.16±9.03 | 4.871 | 0.004 | 2.807 | 0.048 | 0.009 | 0.923 |
|  | healthy control group（n=56） | 69.29±10.47 | 69.45±10.60 | 68.88±11.64 | 67.70±9.55 |  |  |  |  |  |  |
| Notes: PD group: 7-10 days before menstruation (T_0_), maximal pain during menstruation (T_1_), immediately after acupuncture analgesia (T_2_), and 30 mins after acupuncture analgesia(T_3_). healthy control group: 7-10 days before menstruation(T_0_), the first-second days during menstruation (T_1_), 30 mins after T_1_ (T_2_), and 30 mins after T_2_ (T_3_).  ★P< 0.05, compared with PD group. | | | | | | | | | | | |

Table 5 Comparison of changes in pulse-graph measurements between CCBSP and QSBSP at four time points (`x±s)

|  | |  | | Observation Time | | | | | | | | Time effect | | | Interaction  effect | | | | Group effect | | | |
| --- | --- | --- | --- | --- | --- | --- | --- | --- | --- | --- | --- | --- | --- | --- | --- | --- | --- | --- | --- | --- | --- | --- |
| Items(units) | | Patterns(Cases) | | T_0_ | | T_1_ | | T_2_ | | T_3_ | | *F* | | *P* | *F* | | *P* | | *F* | | *P* | |
| h_1(mm)_ | CCBSP(n=54) | | 13.61±4.21 | | 14.53±4.04 | | 15.22±4.42 | | 15.07±3.82 | | 4.76 | | 0.003 | | | 0.213 | | 0.888 | | 0.422 | | 0.518 |
|  | QSBSP(n=37) | | 14.43±4.07 | | 14.74±3.98 | | 15.53±4.23 | | 15.56±3.52 | |  | |  | | |  | |  | |  | |  |
| h_2_(mm) | CCBSP(n=54) | | 9.61±3.00 | | 11.20±2.90 | | 10.64±3.26 | | 11.20±3.11 | | 7.44 | | 0.00 | | | 0.53 | | 0.66 | | 0.000 | | 1.00 |
|  | QSBSP(n=37) | | 10.01±2.78 | | 10.99±2.93 | | 10.74±2.80 | | 10.91±2.47 | |  | |  | | |  | |  | |  | |  |
| h_3_(mm) | CCBSP(n=54) | | 9.38±2.89 | | 11.06±2.98 | | 10.69±3.20 | | 11.01±3.16 | | 7.448 | | 0 | | | 0.949 | | 0.417 | | 0.03 | | 0.87 |
|  | QSBSP(n=37) | | 10.03±2.82 | | 10.86±2.96 | | 10.87±3.07 | | 10.74±2.48 | |  | |  | | |  | |  | |  | |  |
| h_4_(mm) | CCBSP(n=54) | | 5.65±1.93 | | 6.68±1.99 | | 6.92±2.27 | | 7.12±2.45 | | 5.676 | | 0.001 | | | 2.237 | | 0.084 | | 0.027 | | 0.871 |
|  | QSBSP(n=37) | | 6.29±2.14 | | 6.63±2.50 | | 6.64±2.15 | | 6.58±2.10 | |  | |  | | |  | |  | |  | |  |
| h_5(mm)_ | CCBSP(n=54) | | 0.90±0.93 | | 0.55±0.96 | | 0.75±0.84 | | 0.61±0.79 | | 1.525 | | 0.212 | | | 1.112 | | 0.342 | | 2.809 | | 0.097 |
|  | QSBSP(n=37) | | 0.96±10.25 | | 0.88±10.23 | | 10.03±0.91 | | 10.06±10.10 | |  | |  | | |  | |  | |  | |  |
| t_1_(s) | CCBSP(n=54) | | 0.12±0.02 | | 0.13±0.02 | | 0.12±0.02 | | 0.12±0.01 | | 3.805 | | 0.012 | | | 3.18 | | 0.026 | | 2.625 | | 0.107 |
|  | QSBSP(n=37) | | 0.12±0.02 | | 0.12±0.01◆ | | 0.12±0.02 | | 0.12±0.02 | |  | |  | | |  | |  | |  | |  |
| t_2_(s) | CCBSP(n=54) | | 0.21±0.03 | | 0.21±0.03 | | 0.21±0.03 | | 0.21±0.03 | | 0.095 | | 0.963 | | | 0.756 | | 0.52 | | 4.19 | | 0.04 |
|  | QSBSP(n=37) | | 0.20±0.02 | | 0.20±0.02 | | 0.20±0.02 | | 0.21±0.03 | |  | |  | | |  | |  | |  | |  |
| t_3_(s) | CCBSP(n=54) | | 0.24±0.04 | | 0.24±0.04 | | 0.24±0.04 | | 0.24±0.05 | | 0.099 | | 0.961 | | | 0.32 | | 0.811 | | 2.80 | | 0.10 |
|  | QSBSP(n=37) | | 0.23±0.04 | | 0.23±0.02 | | 0.23±0.03 | | 0.23±0.04 | |  | |  | | |  | |  | |  | |  |
| t_4_(s) | CCBSP(n=54) | | 0.36±0.04 | | 0.37±0.06 | | 0.35±0.07 | | 0.34±0.06 | | 1.957 | | 0.128 | | | 1.68 | | 0.178 | | 0.936 | | 0.336 |
|  | QSBSP(n=37) | | 0.33±0.03 | | 0.35±0.040 | | 0.36±0.07 | | 0.35±0.04 | |  | |  | | |  | |  | |  | |  |
| t_5_(s) | CCBSP(n=54) | | 0.53±0.12 | | 0.54±0.12 | | 0.61±0.13 | | 0.58±0.15 | | 5.65 | | 0.00 | | | 2.86 | | 0.04 | | 0.61 | | 0.44 |
|  | QSBSP(n=37) | | 0.55±0.12 | | 0.54±0.10 | | 0.57±0.12 | | 0.53±0.11 | |  | |  | | |  | |  | |  | |  |
| t(s) | CCBSP(n=54) | | 0.88±0.13 | | 0.91±0.12 | | 0.97±0.14 | | 0.92±0.15 | | 6.28 | | 0.00 | | | 1.22 | | 0.30 | | 0.07 | | 0.80 |
|  | QSBSP(n=37) | | 0.91±0.14 | | 0.90±0.13 | | 0.94±0.11 | | 0.91±0.14 | |  | |  | | |  | |  | |  | |  |
| t_1_/t(-) | CCBSP(n=54) | | 0.14±0.03 | | 0.14±0.03 | | 0.12±0.02 | | 0.14±0.03 | | 9.986 | | 0 | | | 1.769 | | 0.153 | | 0.692 | | 0.408 |
|  | QSBSP(n=37) | | 0.13±0.02 | | 0.14±0.02 | | 0.13±0.02 | | 0.14±0.02 | |  | |  | | |  | |  | |  | |  |
| t_4_/t_5_(-) | CCBSP(n=54) | | 0.71±0.21 | | 0.73±0.25 | | 0.62±0.24 | | 0.65±0.22 | | 1.22 | | 0.30 | | | 3.08 | | 0.03 | | 0.06 | | 0.82 |
|  | QSBSP(n=37) | | 0.63±0.12◆ | | 0.68±0.14 | | 0.67±0.33 | | 0.68±0.14 | |  | |  | | |  | |  | |  | |  |
| w_1_ | CCBSP(n=54) | | 0.17±0.07 | | 0.20±0.06 | | 0.16±0.06 | | 0.18±0.07 | | 7.686 | | 0 | | | 0.911 | | 0.436 | | 0.507 | | 0.478 |
|  | QSBSP(n=37) | | 0.15±0.05 | | 0.20±0.08 | | 0.17±0.08 | | 0.17±0.08 | |  | |  | | |  | |  | |  | |  |
| w_1_/t(-) | CCBSP(n=54) | | 0.20±0.08 | | 0.22±0.06 | | 0.17±0.06 | | 0.20±0.08 | | 6.09 | | 0.00 | | | 1.87 | | 0.14 | | 0.65 | | 0.42 |
|  | QSBSP(n=37) | | 0.17±0.05 | | 0.22±0.08 | | 0.18±0.08 | | 0.19±0.08 | |  | |  | | |  | |  | |  | |  |
| HR(次/min) | CCBSP(n=54) | | 70.61±9.77 | | 67.78±9.09 | | 65.61±8.63 | | 67.57±9.56 | | 9.21 | | 0.00 | | | 0.30 | | 0.80 | | 1.47 | | 0.23 |
|  | QSBSP(n=37) | | 72.03±9.03 | | 70.00±8.08 | | 68.88±11.64 | | 69.03±8.25 | |  | |  | | |  | |  | |  | |  |
| Notes: PD group: 7-10 days before menstruation (T_0_), maximal pain during menstruation (T_1_), immediately after acupuncture analgesia (T_2_), and 30 mins after acupuncture analgesia(T_3_).  Notes: ◆P< 0.05, compared with CCBSP | | | | | | | | | | | | | | | | | | | | | | |
